# Orangutans Not Infected with *Plasmodium vivax* or *P. cynomolgi*, Indonesia

**DOI:** 10.3201/eid1510.090364

**Published:** 2009-10

**Authors:** Balbir Singh, Paul Cliff Simon Divis

**Affiliations:** Universiti Malaysia Sarawak, Kuching, Sarawak, Malaysia

**Keywords:** Orangutans, malaria, Plasmodium vivax, Plasmodium cynomolgi, parasites, Indonesia, dispatch

## Abstract

After orangutans in Indonesia were reported as infected with *Plasmodium cynomolgi* and *P. vivax*, we conducted phylogenetic analyses of small subunit ribosomal RNA gene sequences of *Plasmodium* spp. We found that these orangutans are not hosts of *P. cynomolgi* and *P. vivax.* Analysis of >1 genes is needed to identify *Plasmodium* spp. infecting orangutans.

Parasites belonging to the genus *Plasmodium* cause malaria and are usually host specific. For example, humans are natural hosts for *P. falciparum, P. vivax, P. malaria,* and *P. ovale*, and orangutans are naturally infected with *P. pitheci* and *P. silvaticum* (*1*,*2*). However, simian malaria parasites can infect humans (*1*); for example, *P. knowlesi*, normally associated with infections in long-tailed and pig-tailed macaques, has recently been found to have caused malaria in humans in several countries in Southeast Asia (*3*–*8*). This finding raises the possibility that other zoonotic malaria parasites may emerge in humans.

Malaria parasites have distinct small subunit ribosomal RNA (SSU rRNA) genes that are developmentally regulated (*9*). Each *Plasmodium* species has at least 2 types of SSU rRNA genes, and the stage-specific expression of these genes varies among species. In general, the A-type genes are transcribed or expressed mainly during the asexual stages, and the S-type genes are transcribed mainly during the sporozoite stage. *P. vivax* also has O-type genes, which are expressed during ookinete and oocyst development. Phylogenetic analysis of the *P. vivax* and *P. cynomolgi* SSU rRNA genes has indicated that the genes appear to have evolved as a result of 2 gene duplication events (*10*). A more recent study, involving SSU rRNA sequence data from a much larger number of *Plasmodium* spp., demonstrated that gene duplication events giving rise to the A-type and S-type sequences took place independently at least 3 times during the evolution of *Plasmodium* spp. (*11*).

Reid et al. (*12*) analyzed the DNA sequences of SSU rRNA genes of *Plasmodium* spp. from blood of orangutans in Kalimantan, Indonesia. Using phylogenetic analysis, they concluded that, in addition to *P. pitheci* and *P. silvaticum*, the orangutans were infected with the human malaria parasite *P. vivax* and the macaque malaria parasite *P. cynomolgi*. Their report implies that human and macaque malaria parasites could be transmitted to orangutans and that orangutans could act as reservoir hosts for at least 1 of the human malaria parasites.

When taxonomic inferences of species within a genus are made from phylogenetic trees, trees must be reconstructed by using orthologous genes and must include as many species sequences as possible. However, Reid et al. used sequence data of only the S-type SSU rRNA genes for *P. vivax, P. cynomolgi,* and *P. knowlesi* and data of only the A-type genes for *P. inui* and *P. fragile*. Furthermore, they analyzed sequence data from only 4 simian malaria parasites. Nishimoto et al. recently included data from 10 simian malaria parasites (*11*). We therefore reanalyzed the SSU rRNA sequence data of malaria parasites of orangutans together with the A-type, S-type, and O-type SSUrRNA gene sequence data for various *Plasmodium* spp.

## The Study

We used the neighbor-joining method, as described previously, to reconstruct the phylogenetic tree (*3*). Our phylogenetic analyses showed that SSU rRNA gene sequences VM88, VM82, and VM40 from orangutans (*12*) represent A-type SSU rRNA genes and that the VS63 sequence represents an S-type gene of *Plasmodium* spp. (Figure). No morphologic features of the malaria parasite stages in the blood were described for the Kalimantan orangutans by Reid et al. (*12*)*.* Therefore, on the basis of SSU rRNA sequence data available for VM82 and VM88, whether these represent *P. pitheci* or *P. silvaticum*, previously described malaria parasites of orangutans, or some other species of *Plasmodium* cannot be determined with certainty.

**Figure:**
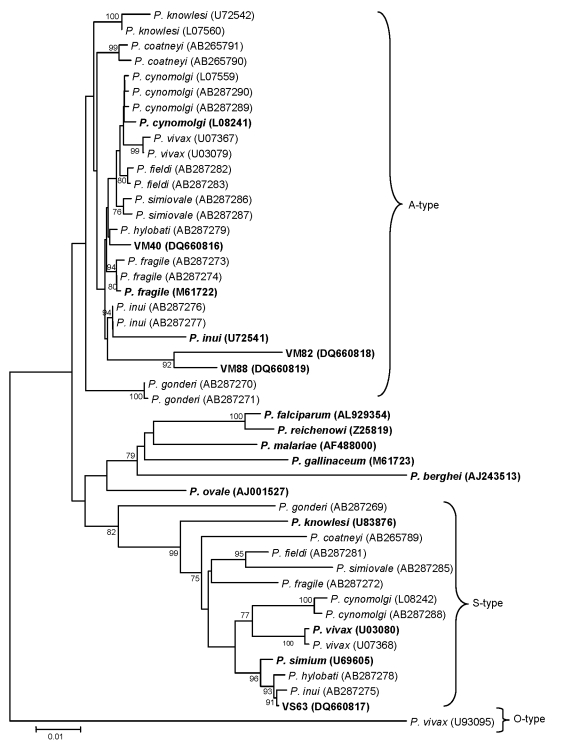
Phylogenetic relationship of *Plasmodium* spp. inferred from small subunit ribosomal RNA sequences. Tree was reconstructed by using the neighbor-joining method. **Boldface** indicates those sequences derived from orangutans (VM40, VM82, VM88, and VS63) and those used by Reid et al. (*12*) in their phylogenetic analysis. Numerals on the branches are bootstrap percentages based on 1,000 replicates; only those >70% are shown. GenBank accession numbers are in brackets. Scale bar indicates nucleotide substitutions per site.

The VS63 sequence is clearly not *P. vivax,* as previously reported by Reid et al. (*12*); it represents a *Plasmodium* sp. that is closely related to *P. inui*. It is most probably the S-type gene for either VM82 or VM88, which are A-type genes of *P. pitheci* and/or *P. silvaticum*. Furthermore, the VM40 sequence from orangutans represents a *Plasmodium* sp. closely related to the gibbon malaria parasite, *P. hylobati (1)*, and is not the macaque malaria parasite, *P. cynomolgi,* as previously reported (*12*)*.*

## Conclusions

Phylogenetic analyses of the SSU rRNA genes indicate that none of the *Plasmodium* spp. isolated from orangutans in Kalimantan, Indonesia, are *P. cynomolgi* or *P. vivax*, as previously reported by Reid et al. (*12*)*.* Before any conclusion about the identity of the malaria parasites infecting orangutans and their corresponding SSU rRNA gene sequences can be derived, a second or third gene of malaria parasites from these orangutans needs to be analyzed and the morphology of the corresponding blood stages needs to be described. Our study underscores the importance of using orthologous genes and sequence data from as many species as possible when inferring species within a genus from phylogenetic trees.
